# Novel Structurally Related Flavones Augment Cell Death Induced by rhsTRAIL

**DOI:** 10.3390/ijms18061211

**Published:** 2017-06-06

**Authors:** Joanna Bronikowska, Ewelina Szliszka, Edyta Kostrzewa-Susłow, Dagmara Jaworska, Zenon P. Czuba, Piotr Bednarski, Wojciech Król

**Affiliations:** 1 Department of Microbiology and Immunology, School of Medicine with the Division of Dentistry in Zabrze, Medical University of Silesia in Katowice, Jordana 19, Zabrze 41808, Poland; eszliszka@sum.edu.pl (E.S.); djaworska@sum.edu.pl (D.J.); zczuba@sum.edu.pl (Z.P.C.); wkrol56@gmail.com (W.K.); 2 Department of Chemistry, Wrocław University of Environmental and Life Sciences, Norwida 25, Wrocław 50375, Poland; ekostrzew@gmail.com; 3 Institute of Rheumatology in Warsaw, Spartańska 1, Warszawa 02637, Poland; piotrbednarski@post.pl

**Keywords:** flavones, cytotoxicity, apoptosis, TRAIL, cancer cells

## Abstract

TRAIL (tumor necrosis factor-related apoptosis-inducing ligand) was identified as a powerful activator of apoptosis in tumor cells and one of the most promising candidates for cancer therapy with no toxicity against normal tissues. However, many tumor cells are resistant to TRAIL-induced apoptosis. The aim of this work was to analyze the improvement of the anticancer effect of rhsTRAIL (recombinant human soluble TRAIL) by nine flavones: 5-Hydroxyflavone, 6-Hydroxyflavone, 7-Hydroxyflavone and their new synthetic derivatives 5-acetoxyflavone, 5-butyryloxyflavone, 6-acetoxyflavone, 6-butyryloxyflavone, 7-acetoxyflavone and 7-butyryloxyflavone. We examined the cytotoxic and apoptotic effects of rhsTRAIL enhanced by novel structurally-related flavones on SW480 and SW620 colon cancer cells using the 3-(4,5-dimethylthiazol-2-yl)-2,5-diphenyltetrazolium bromide test, the lactate dehydrogenase assay and annexin V-FITC fluorescence staining. We observed a slight difference in the activities of the flavones that was dependent on their chemical structure. Our study indicates that all nine flavones significantly augment cell death by rhsTRAIL (cytotoxicity range 36.8 ± 1.7%–91.4 ± 1.7%; apoptosis increase of 33.0 ± 0.7%–78.5 ± 0.9%). Our study demonstrates the potential use of tested flavones in TRAIL-based anticancer therapy and prevention.

## 1. Introduction

Flavones are polyphenolic compounds of the flavonoid class. Natural or synthetic flavones possess antioxidant, antimicrobial, anti-inflammatory, chemopreventive and/or anticancer properties and therefore have beneficial effects on health [1,2,3,4,5].

Because biochemical action depends on the individual flavonoid structure, each compound should be evaluated systematically to assess its individual biological potency. Accordingly, the anticancer effects of flavone analogs alone and in combination with tumor necrosis factor-related apoptosis-inducing ligand (TRAIL) were analyzed. 

Numerous studies have reported interesting properties of flavonoids in anticancer strategies, with notable pleiotropic influences on immune and cancer cells [6]. Flavones exhibit chemopreventive and antitumor actions by interfering with apoptosis or proliferation signaling and regulating the immune system [1,7,8]. TRAIL, a TNF (tumor necrosis factor) superfamily member, is considered to be an endogenous anticancer agent because of its selective cytotoxicity against tumor cells compared to normal primary cells [9,10]. This death ligand is expressed on T lymphocytes, natural killer cells, neutrophils, monocytes and macrophages [7,11]. Membrane-bound TRAIL can be cleaved from the cell surface into a soluble secreted form. Whether soluble or expressed on immune cells, TRAIL molecules play an important role in the surveillance and elimination of tumors [12,13]. The growing interest in TRAIL-based interventions has led to the development of recombinant human TRAIL (rhTRAIL) as a promising therapy for different types of human cancer [14]. The preliminary results of phase I and II clinical trials on rhTRAIL in patients with advanced neoplastic diseases indicate that the direction of these studies is proper. However, some types of cancer cells are resistant to TRAIL-mediated apoptosis [15,16]. The induction of cancer cell-specific apoptosis via enhanced TRAIL signaling has become an important focus of cancer research [17,18]. Most commonly-used standard chemotherapeutic agents including gemcitabine, irinotecan, doxorubicin, 5-fluorouracil and cisplatin, as well as natural or new synthetic compounds have been shown to sensitize cancer cells to TRAIL-mediated apoptosis [7,19]. Various studies confirm that flavones also augment the antitumor activity of TRAIL and overcome TRAIL resistance in cancer cells [7] and suggest a significant role for various natural flavones in anticancer immune effector mechanisms [7,20].

**Figure 1:**
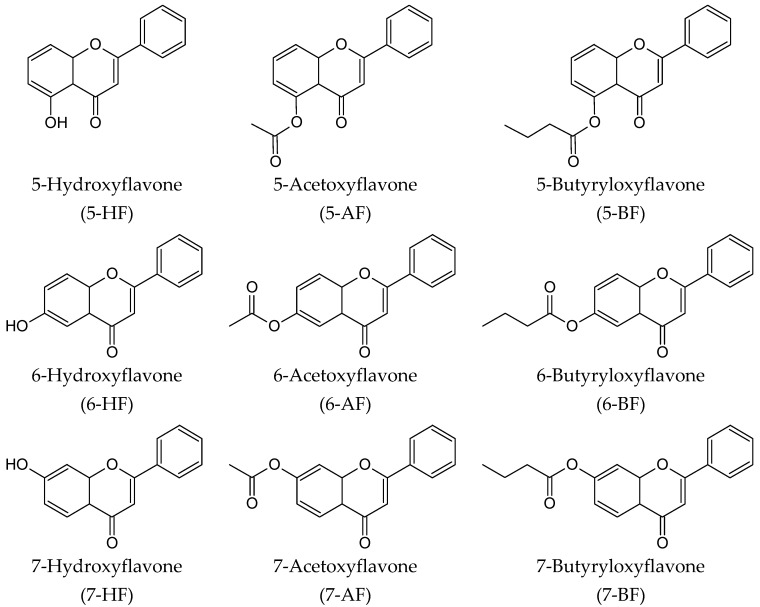
Chemical structures of the tested flavones. HF, Hydroxyflavone; AF, Acetoxyflavone; BF, Butyryloxyflavone.

In the present study, we examined the cytotoxic and apoptotic effects of recombinant human soluble TRAIL (rhsTRAIL) in combination with nine natural and synthetic flavones: 5-Hydroxyflavone (5-HF) and its derivatives, 5-acetoxyflavone (5-AF) and 5-butyryloxyflavone (5-BF), 6-Hydroxyflavone (6-HF) and its derivatives, 6-acetoxyflavone (6-AF) and 6-butyryloxyflavone (6-BF), and 7-Hydroxyflavone (7-HF) and its derivatives, 7-acetoxyflavone (7-AF) and 7-butyryloxyflavone (7-BF) (Figure 1) in SW480 and SW620 colon cancer cells.

In our work, we describe for the first time the enhancement of rhsTRAIL-mediated apoptosis in cancer cells by nine flavones: 5-HF, 6-HF, 7-HF and their new synthetic derivatives with an acetyl or a butyryl group.

## 2. Results

### 2.1. Cytotoxic and Apoptotic Effects of Flavones in Colon Cancer Cells

**Figure 2:**
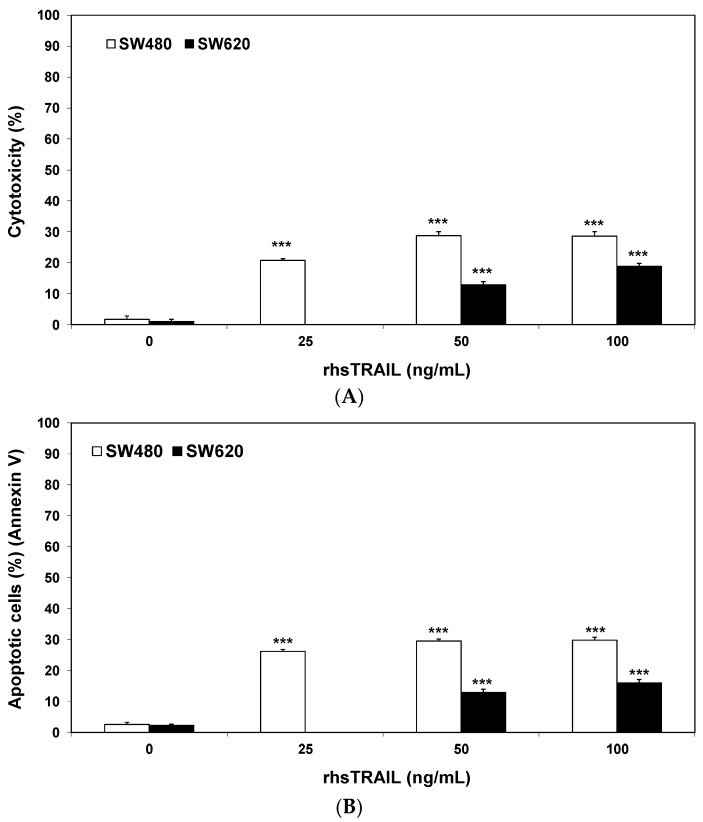
Cytotoxic and apoptotic effects of rhsTRAIL on SW480 and SW620 colon cancer cells. Cells were incubated with 25–100 ng/mL TRAIL for 48 h. The values represent the mean ± SD of three independent experiments (*n* = 3). (**A**) Cytotoxic activity of rhsTRAIL against colon cancer cells. The percentage of cell death was measured using the MTT cytotoxicity assay (*** *p* < 0.001 compared to control without rhsTRAIL); (**B**) Apoptotic activity of rhsTRAIL against colon cancer cells. Apoptotic cell death was detected by flow cytometry using annexin V-FITC staining (*** *p* < 0.001 compared to control without rhsTRAIL).

**Figure 3:**
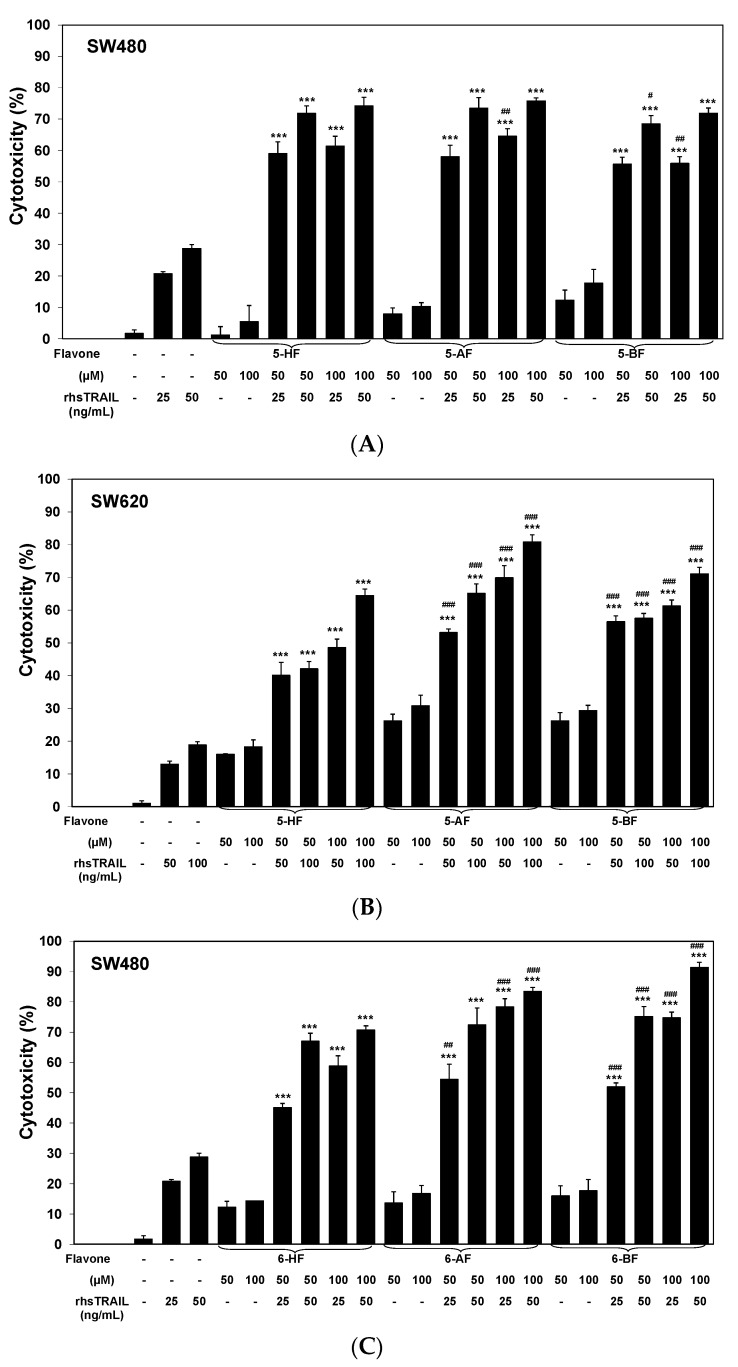

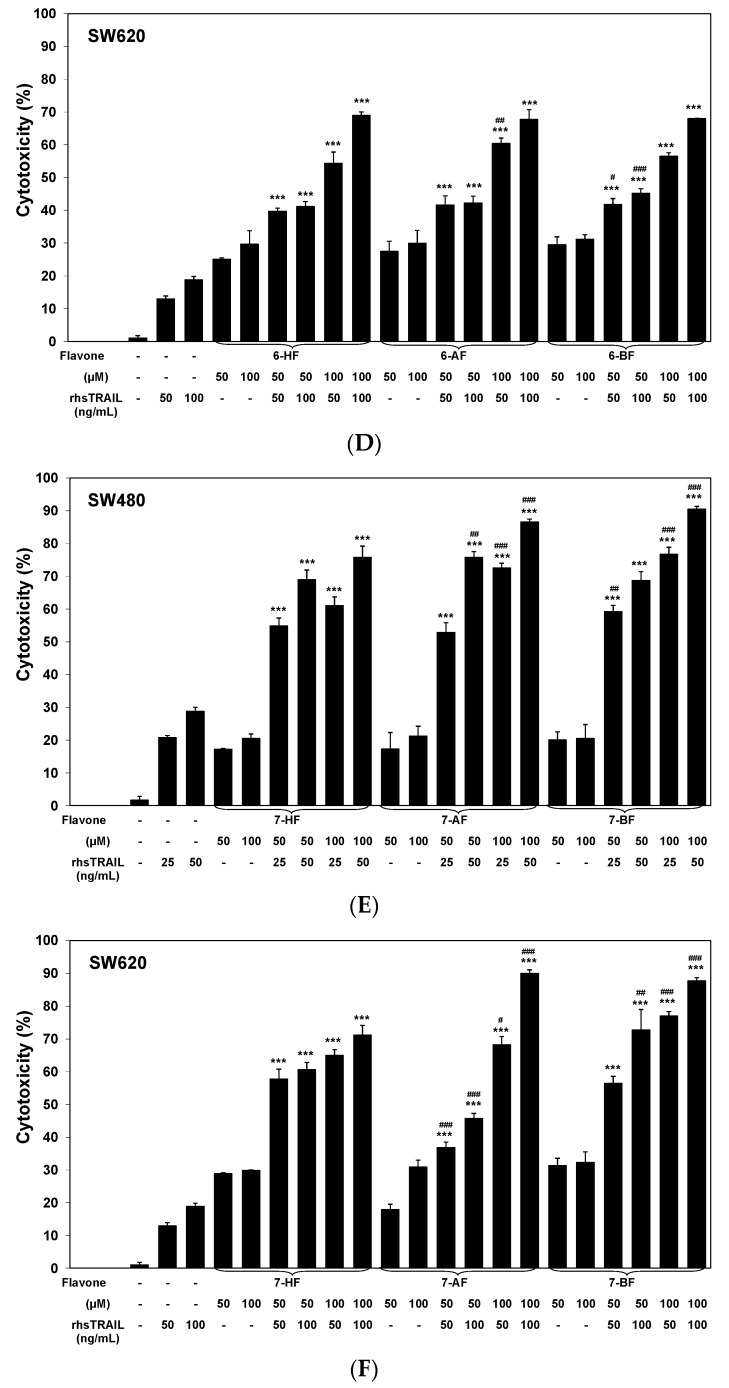
Cytotoxic effect of rhsTRAIL in combination with flavones on SW480 and SW620 colon cancer cells. Cells were incubated with rhsTRAIL at concentrations of 25–100 ng/mL and/or the compounds at 50 μM and 100 μM for 48 h. The values represent the mean ± SD of three independent experiments (*n* = 3). The percentage of cell death was measured using the MTT cytotoxicity assay (*** *p* < 0.001 compared to rhsTRAIL, ^#^ *p* < 0.05, ^##^ *p* < 0.01 and ^###^ *p* < 0.001 compared to rhsTRAIL + substrate: 5-HF or 6-HF or 7-HF). Cytotoxic activity of rhsTRAIL with flavones: (**A**) 5-HF, 5-AF or 5-BF against SW480 cells; (**B**) 5-HF, 5-AF or 5-BF against SW620 cells; (**C**) 6-HF, 6-AF or 6-BF against SW480 cells; (**D**) 6-HF, 6-AF or 6-BF against SW620 cells; (**E**) 7-HF, 7-AF or 7-BF against SW480; and (**F**) 7-HF, 7-AF or 7-BF against SW620 cells.

Various in vitro and in vivo studies confirm the anticancer activity of flavones [1,21,22,23]. In addition to two natural flavones (5-HF (also known as primuletin) and 7-HF), another seven analogs (5-AF, 5-BF, 6-HF, 6-AF, 6-BF, 7-AF, 7-BF) are synthetic compounds. First, we examined the cytotoxicity and apoptosis induced by nine flavones, 5-HF, 6-HF, 7-HF and their new synthetic derivatives (5-AF, 5-BF, 6-AF, 6-BF, 7-AF, 7-BF) at concentrations of 50 μM and 100 μM in SW480 and SW620 colon cancer cells (Figure 2 and Figure 3). Flavones cause their cytotoxic effect in colon cancer cells via the apoptotic route. The necrotic cell death percentage of cancer cells examined by the lactate dehydrogenase (LDH) assay and flow cytometry with propidium iodide was near 0%. 

The activity of the flavones was dependent on the dose and structure of the compound and on the tested cell line, with 7-HF and its two analogs at 50 µM and 100 μM possessing the strongest anticancer properties (Supplementary Figures S1 and S2). The obtained data indicate higher activity of the tested flavones against SW620 than SW480. 

A similar or slightly weaker activity against SW480 and SW620 colon cancer cells was exhibited by 6-HF and its analogs at the concentrations of 50 μM and 100 μM. 6-HF, 6-AF and 6-BF caused higher cell death in SW620 cells than in SW480 cells. The apoptosis induced by these flavones was 10.3 ± 0.9%–15.6 ± 0.8% in SW480 cells and 21.4 ± 0.5%–26.4 ± 0.5% in SW620 cells. Although 5-HF at 50 μM and 100 μM caused a weak anticancer effect (1.2 ± 2.6%–5.4 ± 5.2% cytotoxicity and 3.6 ± 0.5%–5.8 ± 0.6% apoptosis in SW480 cells, 16.2 ± 0.2%–18.3 ± 2.1% cytotoxicity and 13.3 ± 0.6%–16.0 ± 0.6% apoptosis in SW620 cells), the cytotoxicity and apoptosis triggered by 5-AF and 5-BF was higher compared to their precursor (7.9 ± 1.9%–17.7 ± 4.4% cytotoxicity and 6.9 ± 0.6%–14.6 ± 0.8% apoptosis in SW480 cells, 26.2 ± 2.5%–30.8 ± 3.2% cytotoxicity and 21.4 ± 0.9%–26.2 ± 0.5% apoptosis in SW620 cells) (Supplementary Figures S1 and S2).

The obtained results suggest that a hydroxyl group located at the C6 or C7 position, an acetoxyl group located at the C6 or C7 position (and also C5 position for SW620) and a butyryl group located at the position C5, or C6, or C7 determines the strength of the cytotoxic and apoptotic effects of the compounds against colon cancer cells. We observed differences in the sensitivity of the malignant cell lines in our study; in contrast to SW480 cells, SW620 cells were more susceptible to the anticancer activity of flavones.

### 2.2. Cytotoxic and Apoptotic Effects of TRAIL in Combination with Flavones in Colon Cancer Cells

**Figure 4:**
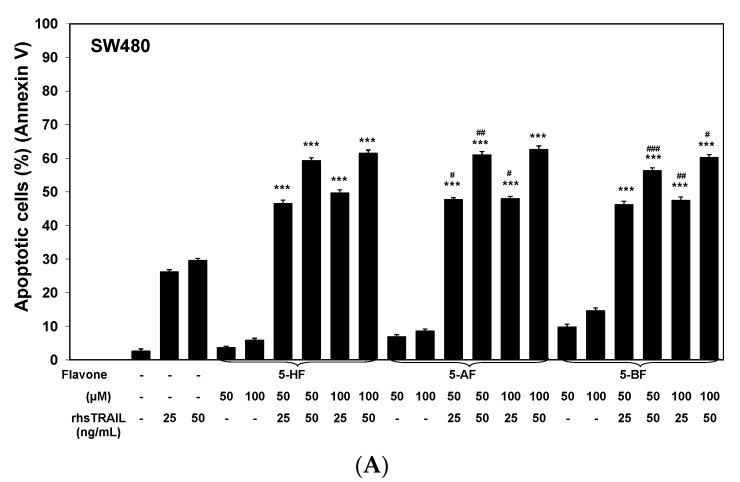

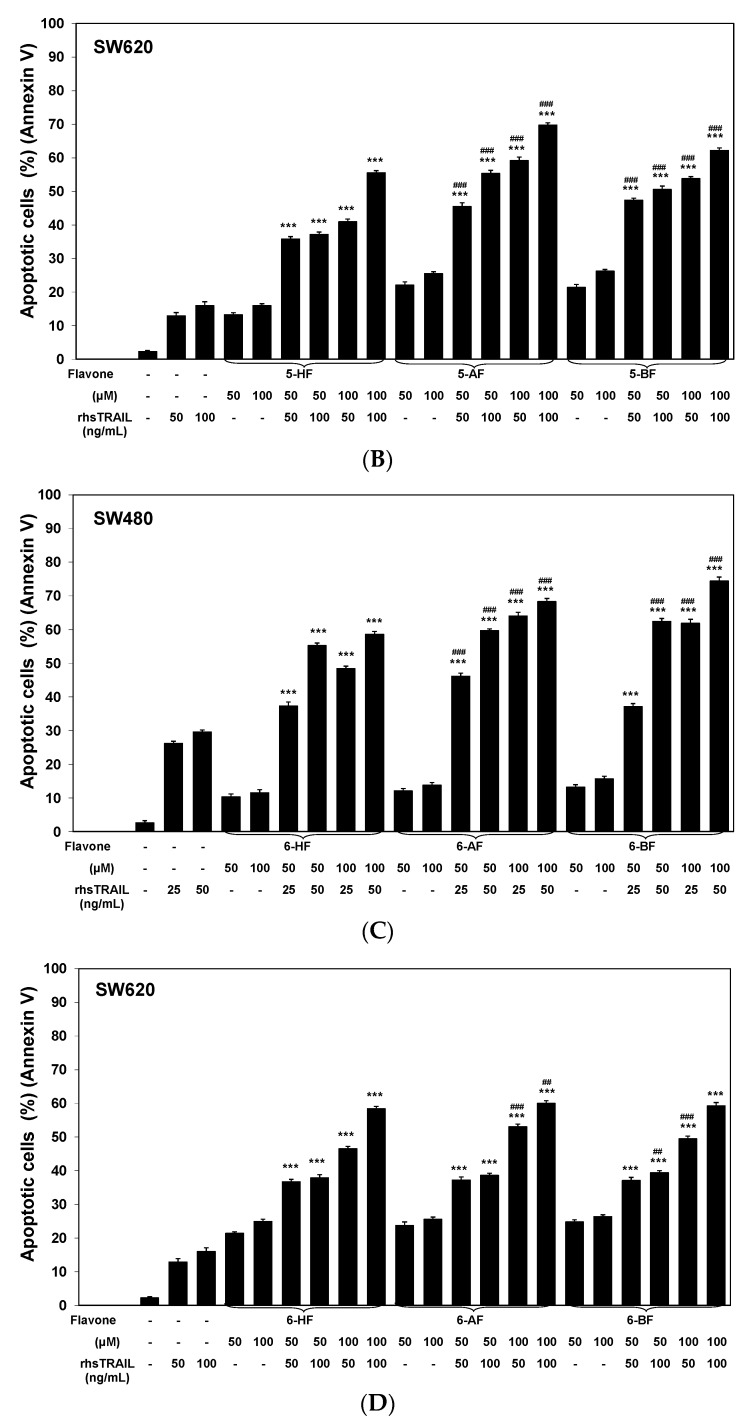

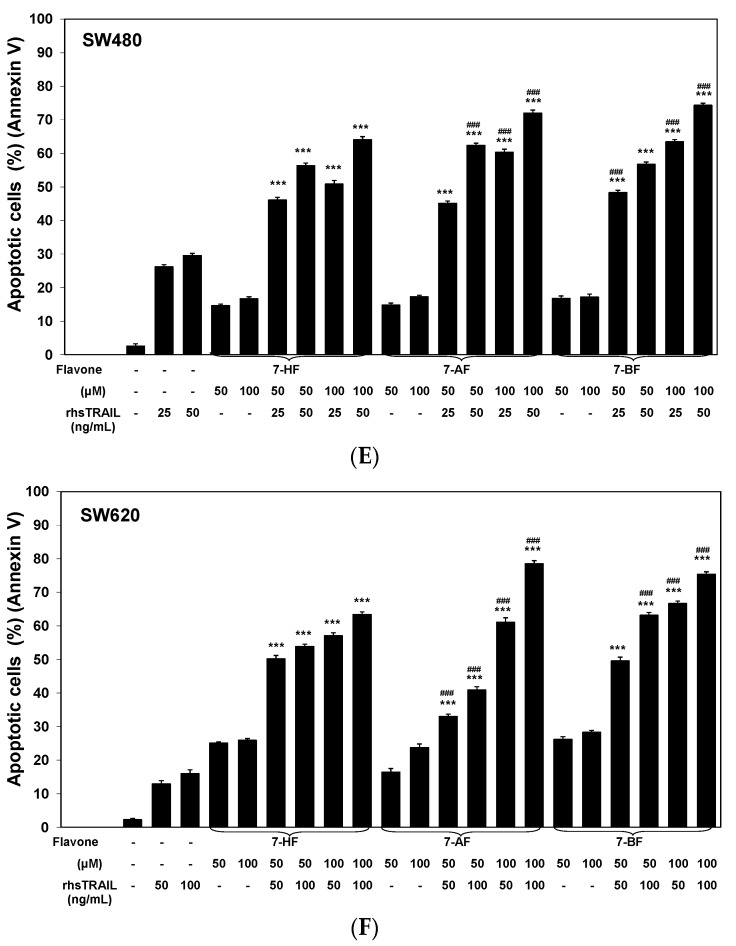
Apoptotic effect of rhsTRAIL in combination with flavones on SW480 and SW620 colon cancer cells. Cells were incubated with rhsTRAIL at concentrations of 25–100 ng/mL and/or the compounds at concentrations of 50 μM and 100 μM for 48 h. The values represent the mean ± SD of three independent experiments performed in duplicate (*n* = 6). Apoptotic cell death was detected by flow cytometry using annexin V-FITC staining (*** *p* < 0.001 compared to rhsTRAIL, ^#^ *p* < 0.05, ^##^ *p* < 0.01 and ^###^ *p* < 0.001 compared to rhsTRAIL + substrate). Apoptotic activity of rhsTRAIL with flavones: (**A**) 5-HF, 5-AF or 5-BF against SW480 cells; (**B**) 5-HF, 5-AF or 5-BF against SW620 cells; (**C**) 6-HF, 6-AF or 6-BF against SW480 cell; (**D**) 6-HF, 6-AF or 6-BF against SW620 cells; (**E**) 7-HF, 7-AF or 7-BF against SW480; and (**F**) 7-HF, 7-AF or 7-BF against SW620 cells.

The rhsTRAIL used in our study is a soluble protein based on a natural endogenous ligand [14,24]. We first tested the anticancer effect of rhsTRAIL on both colon cancer cell lines (Figure 4). The cell death induced by 25–100 ng/mL TRAIL in the SW480 cell line reached 20.8 ± 0.6%–28.7 ± 1.3% and rhsTRAIL at concentrations of 50–100 ng/mL caused 12.9 ± 1.0%–18.8 ± 1.0% cell death in the SW620 cell line. The necrotic cell death percentage of cancer cells revealed by an LDH assay and flow cytometry with propidium iodide was near 0%. rhsTRAIL at the same concentration triggered apoptosis in 26.2 ± 0.7%–29.8 ± 0.9% of SW480 cells and in 12.9 ± 1.0%–16.0 ± 1.1% of SW620 cells. rhsTRAIL at concentrations of 200 ng/mL or higher did not significantly increase this anticancer effect on the cancer cells (data not shown).

We confirmed that both colon cancer cell lines are resistant to rhsTRAIL-induced apoptosis. The SW480 cell line was more susceptible to the anticancer activity of rhsTRAIL than the SW620 cell line. Therefore, in further studies, we used rhsTRAIL at 25 ng/mL and 50 ng/mL for the SW480 cells and 50 ng/mL and 100 ng/mL for the SW620 cells. 

The cytotoxicity of the combination of 50 ng/mL TRAIL with 100 μM flavone in SW480 cell line was 83.5 ± 1.3% cell death for 6-AF, 91.4 ± 1.7% cell death for 6-BF, 86.6 ± 0.9% cell death for 7-AF and 90.5 ± 0.8% cell death for 7-BF. The cytotoxic effect of 100 ng/mL TRAIL with 100 μM flavone against the SW620 cell line increased to 80.8 ± 2.2% cell death for 5-AF, 71.2 ± 2.9% cell death for 7-HF, 90.0 ± 1.1% cell death for 7-AF and 87.8 ± 1.0% cell death for 7-BF. The cytotoxicity after the co-treatment of TRAIL and flavones is shown in Figure 3.

The TRAIL-mediated apoptotic pathway is a potential target for phenolics and polyphenols [7,25]. Next, the cytotoxic and apoptotic activities of rhsTRAIL in combination with nine flavone analogs at 50 μM and 100 μM on colon cancer cells were investigated. All tested flavones overcame TRAIL resistance in the SW480 and SW620 cells. The combined anticancer action of TRAIL and the tested flavones was dependent on the dose of ligand, the dose and structure of the compound and the type of cell line. The compounds significantly augmented the cytotoxic and apoptotic effects of TRAIL (cytotoxicity increased to 45.1 ± 1.4%–91.4 ± 1.7% in SW480 cells and 36.8 ± 1.7%–90.0 ± 1.1% in SW620 cells; apoptosis increased to 37.1 ± 0.9%–74.4 ± 1.1% in SW480 cells and 33.0 ± 0.7%–78.5 ± 0.9% in SW620 cells).

The co-treatment of rhsTRAIL and the compounds with an acetyl or a butyryl group at position 7 (7-AF, 7-BF) induced significant and the strongest cytotoxicity in SW480 and SW620 cells compared to 7-HF; 7-AF and 7-BF significantly augmented rhsTRAIL-induced cytotoxicity in both colon cancer cell lines compared to 7-HF. 5-AF and 5-BF combined with rhsTRAIL used against SW620 cells and 6-AF and 6-BF combined with rhsTRAIL used against SW480 caused also the strongest cytotoxic activity, as their primary analogs (5-HF and 6-HF, respectively).

Similar results were obtained in measurements of apoptosis for the same concentrations of rhsTRAIL (25–50 ng/mL for SW480 and 50–100 ng/mL for SW620) and flavones (50–100 μM). A flow cytometric analysis using annexin V-FITC and propidium iodide double staining showed the occurrence of the late apoptosis in most cases. rhsTRAIL-induced apoptosis in colon cancer cells after incubation with flavones is demonstrated in Figure 4. The derivatives of 6-HF and 7-HF were potent activators of rhsTRAIL-mediated apoptosis in SW480 cells, with apoptosis at 100 μM flavone and 50 ng/mL TRAIL reaching 68.3 ± 0.9% for 6-AF, 74.4 ± 1.1% for 6-BF, 64.1 ± 0.9% for 7-AF and 74.4 ± 0.6% for 7-BF. 5-AF, 7-HF, 7-AF and 7-BF significantly enhanced rhsTRAIL-mediated apoptosis in SW620 cells; at 100 μM, these compounds increased the apoptotic effect of 100 ng/mL rhsTRAIL to 69.8 ± 0.6% for 5-AF, 63.4 ± 0.8% for 7-HF, 78.5 ± 0.9% for 7-AF and 75.4 ± 0.7% for 7-BF. The necrotic cell death percentage of colon cancer cells incubated with rhsTRAIL and flavones examined by the LDH assay and flow cytometry with propidium iodide was near 0%.

The flavone derivatives with an acetyl or a butyryl group at position 5 (5-AF, 5-BF), at position 6 (6-AF, 6-BF) or at position 7 (7-AF, 7-BF) in combination with rhsTRAIL exhibited a significant higher apoptotic effect against SW480 and SW620 cells compared to their substrates (5-HF, 6-HF, 7-HF).

We observed slight differences between the cytotoxic and apoptotic effect; the cytotoxicity after application of rhsTRAIL and flavones was higher as a result of apoptosis. This indicated that except apoptosis, also other types of cell death could be involved in the cytotoxic effect. 

## 3. Discussion

Previous in vitro studies have shown that various natural or synthetic flavones induce cytotoxicity and apoptosis in tumor cells. Wenzel et al. reported that flavone (2-phenyl-4*H*-1-benzopyran-4-one), the core structure of the flavone subclass, is a potent inhibitor of proliferation and an inducer of apoptosis in cancer cells. Incubation of HCT116 and HT29 colon cancer cells with flavone led to apoptosis and the suppression of cell growth [26]. It has been proven that 6-HF exhibited a growth inhibitory effect in SF295 glioma cells, HCT15 and HCT15CLO2 colon cancer cells, whereas 7-HF did not exert any cytotoxicity against these cell lines [27]. Additionally, 6-HF induced an anti-proliferative effect on ZR751 breast cancer cells and demonstrated apoptotic and anti-proliferative activities against HL60 leukemia cells [28,29]. Chrysin (5,7-diHydroxyflavone) has anti-proliferative and pro-apoptotic effects on PC3 prostate, MDAMB231 breast and HeLa cervical cancer cells [30,31,32]. Apigenin (5,7,4′-triHydroxyflavone) promotes apoptosis and/or cell cycle arrest in T24 bladder, A549 lung, HT29 colon cancer cells and A375 melanoma cells, and baicalein (5,6,7-triHydroxyflavone) causes apoptosis in HT29 colon cancer cells [33,34,35,36,37]. Furthermore, luteolin (5,7,3′,4′-tetraHydroxyflavone) was found to induce apoptosis and block the cell cycle in A549 lung, HCT15 and HT29 colon cancer cells and A375 melanoma cells [38,39,40,41].

We proved that all tested flavone analogs showed a significant anticancer effect. The compounds with a hydroxyl group located at the C6 or C7 position, an acetoxyl group located at the C6 or C7 position (and also the C5 position for SW620) and a butyryl group located at the position C5, or C6, or C7 demonstrated the strong cytotoxic and apoptotic activities against colon cancer cells. In addition, we noticed differences in the sensitivity of the tested colon cell lines; SW620 cells were more susceptible to the anticancer effect of flavones compared to SW480 cells. 

The death ligand TRAIL is a powerful inducer of apoptosis in cancer cells [42]. Endogenous TRAIL expressed on the surface of immune cells or cleaved into a soluble, secreted form plays an important role in the surveillance and defense against malignant tumors [12,13,43,44]. In recent years, numerous exogenous forms of TRAIL have been developed based on the structure and biological activities of the natural ligand. Both pre-clinical and clinical studies with recombinant human soluble TRAIL (rhsTRAIL) have shown a remarkable anticancer effect in a wide range of tumor types [14,15]. However, some cancer cells are resistant to TRAIL-mediated apoptosis [45,46]. The expression of death receptors and proapoptotic or antiapoptotic proteins in cancer cells is involved in TRAIL resistance [15,16,43]. We tested the effect of rhsTRAIL on SW480 and SW620 cells and confirmed that both colon cancer cell lines are resistant to TRAIL-induced apoptosis [45,46]. The SW480 cell line was more susceptible to the anticancer activity of rhsTRAIL than the SW620 cell line.

rhsTRAIL-resistant cancer cells can be sensitized to rhsTRAIL-induced apoptosis with various natural and synthetic flavones [7,47,48]. Next, we examined for the first time the cytotoxic and apoptotic activities of rhsTRAIL in combination with nine flavone analogs on colon cancer cells. The co-treatment effect of TRAIL and flavones was dependent on the concentration of the ligand, on the concentration and structure of the compound and on the type of cell line. All nine flavones significantly augment the anticancer activity of rhsTRAIL. The flavones with a hydroxyl or an acetyl or a butyryl group at position 7 (7-HF, 7-AF, 7-BF) in combination with rhsTRAIL exhibited the highest effect against both colon cancer cell lines. The flavone derivatives with an acetyl or a butyryl group at position 6 (6-AF, 6-BF) and 5-HF in combination with rhsTRAIL exhibited the highest effect against SW480 cells. In contrast, the flavone analogs with an acetyl or a butyryl group at position 5 (5-AF, 5-BF) in combination with TRAIL showed the strongest effect against SW620 cells. 

Numerous studies confirmed that flavones sensitize cancer cells to TRAIL-mediated apoptosis. Indeed, flavone promotes TRAIL-induced apoptosis in HeLa cervical, SW780 and RT112 bladder cancer cells. Chrysin and apigenin have been shown to overcome TRAIL resistance in MDAMB231 breast, Capan1 pancreatic, HeLa cervical, HT29 colon, LNCaP prostate, SW780 and RT112 bladder cancer cells, HepG2 hepatoma cells and SKMEL37 melanoma cells [49,50,51]. Additionally, chrysin potentiates the apoptotic effect of TRAIL in CNE1 nasopharyngeal, HeLa cervical, A549 lung and HCT116 colon cancer cells, and apigenin augments TRAIL-mediated apoptosis in Jurkat leukemia T cells, HeLa cervical, DLD1 colon and DU145 prostate cancer cells [52,53,54]. Baicalein enhances TRAIL-induced death in SW480 colon and PC3 prostate cancer cells [45]. Luteolin reverses TRAIL resistance in CNE1 nasopharyngeal, in HeLa cervical, A549 lung, HT29 colon, SW780 and RT112 bladder, 786O, ACHN and A498 renal cancer cells and HepG2 hepatoma cells [55,56,57,58,59].

TRAIL is considered as an effective inducer of death in cancer cells under clinical investigation. The enhancement of the TRAIL-mediated apoptosis by novel compounds possibly represents a promising strategy.

## 4. Materials and Methods

### 4.1. Chemistry

#### General

^1^H NMR and ^13^C NMR spectra were recorded with a Bruker Avance DRX 300 spectrometer (Bruker BioSpin GmbH, Rheinstetten, Germany). The course of each reaction was monitored by thin layer chromatography (TLC), which was carried out using silica gel 60 F_254_ plates (Merck, Darmstadt, Germany). Chromatograms were developed using hexane:ethyl acetate (7:3) and dichloromethane:ethyl acetate (1:1), and the plates were visualized under UV light. Column chromatography (CC) was performed using the same eluents and silica gel (230–400 mesh, Merck, Darmstadt, Germany). HPLC analyses were performed with a Waters 2690 instrument equipped with a Waters 996 photodiode array detector using an ODS 2 column (4.6 × 250 mm, Waters, Milford, MA, USA) and a Guard-Pak Inserts μBondapak C18 pre-column. The separation conditions were as follows: gradient elution using 80% of acetonitrile in 4.5% formic acid solution (Eluent A) and 4.5% formic acid (Eluent B); flow 1 mL/min; detection wavelength 280 nm; program: 0–7 min, 10% A 90% B; 7–10 min, 50% A 50% B; 10–13 min, 60% A 40% B; 13–15 min, 70% A 30% B; 15–20 min 80% A 20% B; 20–30 min 90% A 10% B; 30–40 min, 100% A. 

The substrates for the reaction were 5-Hydroxyflavone, 6-Hydroxyflavone and 7-Hydroxyflavone. 5-Hydroxyflavone, 6-Hydroxyflavone and 7-Hydroxyflavone were obtained by microbial demethylation of 5-methoxyflavone, 6-methoxyflavone and 7-methoxyflavone, respectively [60,61]. Biotransformations leading to 5-Hydroxyflavone were catalyzed by the enzymatic system of *Aspergillus niger* SBP [60]. Transformations affording 6- and 7-methoxyflavone were conducted using *A. niger* MB strain [61]. Independently, 6-Hydroxyflavone and 7-Hydroxyflavone were obtained by microbial dehydration of 6-Hydroxyflavone and 7-Hydroxyflavone, respectively [62,63,64]. The reactions leading to 6-Hydroxyflavone were catalyzed by the enzymatic system for the strains *A. niger* 13/5 and *A. niger* MB. 7-Hydroxyflavone was obtained by transformation in the culture of the strain *A. niger* 13/5 [63].

5-Hydroxyflavone (5-HF, C_15_H_10_O_3_): R_t_ 20.40 min (HPLC). ^1^H NMR (DMSO-d_6_) δ: 6.84 (1H, d, *J*_6,7_ = 8.2 Hz, H-6), 7.14 (1H, s, H-3), 7.23 (1H, d, *J*_8,7_ = 8.5 Hz, H-8), 7.65 (4H, m, H-3′, H-4′, H-5′, H-7), 8.12 (2H, d, *J* = 7.8 Hz, H-2′, H-6′), 12.7 (1H, s, 5-OH); ^13^C NMR (DMSO-d_6_) δ: 105.6 (C-3), 107.5 (C-8), 110.1 (C-10), 110.9 (C-6), 126.6 (C-2′, C-6′), 129.1 (C-3′, C-5′), 130.5 (C-1′), 132.3 (C-4′), 135.9 (C-7), 155.9 (C-9), 159.8 (C-5), 164.0 (C-2) and 183.2 (C-4) [60].

6-Hydroxyflavone (6-HF, C_15_H_10_O_3_): R_t_ 14.34 min (HPLC). Purity 99% (HPLC). A full description of the ^1^H NMR and ^13^C NMR spectra of 6-HF can be found in our previous paper [61,62,64].

7-Hydroxyflavone (7-HF, C_15_H_10_O_3_): R_t_ 13.72 min (HPLC). Purity 99% (HPLC). A full description of the ^1^H NMR and ^13^C NMR spectra of 7-HF can be found in our previous paper [61,63].

### 4.2. General Procedure for the Esterification of 5-HF, 6-HF and 7-HF

**Scheme 1:**
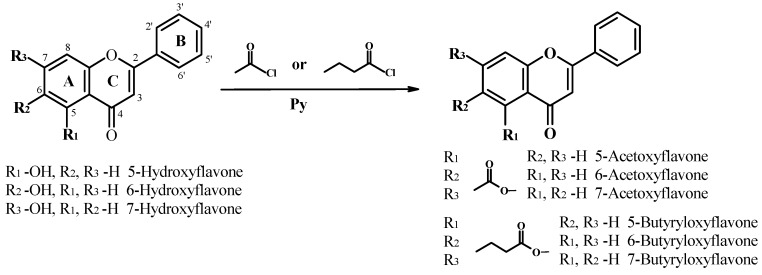
Synthesis of 5-acetoxyflavone, 6-acetoxyflavone, 7-acetoxyflavone and 5-butyryloxyflavone, 6-butyryloxyflavone, 7-butyryloxyflavone.

To 0.25 mmol of 5-HF, 6-HF or 7-HF dissolved in 5 mL of tetrahydrofuran (THF), 0.62 mmol of pyridine and 0.58 mmol of acetyl chloride (butyryl chloride) were added. The reaction mixture was stirred with a magnetic stirrer at room temperature for 30 min (the progress of the reaction was monitored by TLC). When the substrate was fully consumed, 5 mL of ethyl acetate were added, and the reaction mixture was washed with 0.5 M HCl until the solution became slightly acidic (pH = 5). The organic layer was then separated, and the aqueous layer was additionally extracted with ethyl acetate (3 × 5 mL). The combined organic layers were washed with brine until neutral and dried over anhydrous MgSO_4_. The esters were separated by column chromatography (SiO_2_) (Scheme 1). 

Pure esters were identified by spectral analyses (TLC, ^1^H NMR, ^13^C NMR). Spectral data of the products are as follows:

5-Acetoxyflavone (5-AF, C_17_H_12_O_4_): R_t_ 23.52 min (HPLC); purity 99% (HPLC). ^1^H NMR (CDCl_3_) δ: 2.46 (3H, s, CH_3_-), 6.69 (1H, s, H-3), 7.03 (1H, dd, *J*_6,7_ = 7.9 Hz, *J*_6,8_ = 1.0 Hz, H-6), 7.49 (1H, dd, *J*_8,7_ = 8.6 Hz, *J*_8,6_ = 1.1 Hz, H-8), 7.55 (3H, m, H-3′, H-4′, H-5′), 7.67 (1H, dd, *J*_7,8_ = 8.6 Hz, *J*_7,6_ = 7.9 Hz, H-7), 7.91 (2H, m, H-2′, H-6′); ^13^C NMR (CDCl_3_) δ: 21.3 (CH_3_-), 106.2 (C-3), 108.8 (C-8), 116.4 (C-10), 119.4 (C-6), 126.5 (C-2′, C-6′), 129.3 (C-3′, C-5′), 131.9 (C-1′), 133.6 (C-4′), 135.6 (C-7), 149.4 (C-9), 157.6 (C-5), 162.6 (C-2), 170.0 (>C = 0) and 177.3 (C-4).

5-Butyryloxyflavone (5-BF, C_19_H_16_O_4_): R_t_ 27.82 min (HPLC); purity 98% (HPLC). ^1^H NMR (CDCl_3_) δ: 1.09 (3H, t, *J* = 7.5 Hz, CH_3_CH_2_CH_2_-), 1.84 (2H, m, CH_3_CH_2_CH_2_-), 2.74 (2H, t, *J* = 7.5 Hz, CH_3_CH_2_CH_2_-), 6.68 (1H, s, H-3), 7.02 (1H, dd, *J*_6,7_ = 7.9 Hz, *J*_6,8_ = 1.1 Hz, H-6), 7.47 (1H, dd, *J*_8,7_ = 8.5 Hz, *J*_8,6_ = 1.1 Hz, H-8), 7.55 (3H, m, H-3′, H-4′, H-5′), 7.66 (1H, dd, *J*_7,8_ = 8.4 Hz, *J*_7,6_ = 7.9 Hz, H-7), 7.90 (2H, m, H-2′, H-6′); ^13^C NMR (CDCl_3_) δ: 13.8 (CH_3_CH_2_CH_2_-), 18.7 (CH_3_CH_2_CH_2_-), 36.3 (CH_3_CH_2_CH_2_-), 106.2 (C-3), 108.8 (C-8), 116.5 (C-10), 119.5 (C-6), 126.5 (C-2′, C-6′), 129.3 (C-3′, C-5′), 131.9 (C-1′), 133.6 (C-4′), 135.6 (C-7), 149.4 (C-9), 157.8 (C-5), 162.6 (C-2), 172.9 (>C = 0) and 177.4 (C-4).

6-Acetoxyflavone (6-AF, C_17_H_12_O_4_): R_t_ 17.12 min (HPLC); purity 99% (HPLC). ^1^H NMR (CDCl_3_) δ: 2.34 (3H, s, CH_3_-), 6.81 (1H, s, H-3), 7.45 (1H, dd, *J*_7,8_ = 9.0 Hz, *J*_7,5_ = 2.8 Hz, H-7), 7.53 (3H, m, H-3′, H-4′, H-5′), 7.60 (1H, d, *J*_8,7_ = 9.0 Hz, H-8), 7.92 (3H, m, H-2′, H-6′, H-5); ^13^C NMR (CDCl_3_) δ: 21.1 (CH_3_-), 107.3 (C-3), 117.9 (C-5), 119.5 (C-8), 124.9 (C-10), 126.5 (C-2′, C-6′), 128.1 (C-7), 129.2 (C-3′, C-5′), 131.8 (C-1′), 131.9 (C-4′), 147.8 (C-9), 153.9 (C-6), 163.8 (C-2), 169.4 (>C = 0) and 177.8 (C-4).

6-Butyryloxyflavone (6-BF, C_19_H_16_O_4_): R_t_ 21.39 min (HPLC); purity 99% (HPLC). ^1^H NMR (CDCl_3_) δ: 1.07 (3H, t, *J* = 7.4 Hz, CH_3_CH_2_CH_2_-), 1.81 (2H, m, CH_3_CH_2_CH_2_-), 2.58 (2H, t, *J* = 7.4 Hz, CH_3_CH_2_CH_2_-), 6.81 (1H, s, H-3), 7.44 (1H, dd, *J*_7,8_ = 9.0 Hz, *J*_7,5_ = 2.8 Hz, H-7), 7.53 (3H, m, H-3′, H-4′, H-5′), 7.59 (1H, d, *J*_8,7_ = 9.0 Hz, H-8), 7.92 (3H, m, H-2′, H-6′, H-5); ^13^C NMR (CDCl_3_) δ: 13.8 (CH_3_CH_2_CH_2_-), 18.6 (CH_3_CH_2_CH_2_-), 36.2 (CH_3_CH_2_CH_2_-), 107.3 (C-3), 117.9 (C-5), 119.5 (C-8), 124.9 (C-10), 126.5 (C-2′, C-6′), 128.1 (C-7), 129.2 (C-3′, C-5′), 131.8 (C-1′), 131.9 (C-4′), 147.9 (C-9), 153.8 (C-6), 163.8 (C-2), 172.1 (>C = 0) and 177.9 (C-4).

7-Acetoxyflavone (7-AF, C_17_H_12_O_4_): R_t_ 17.21 min (HPLC); purity 99% (HPLC).^1^H NMR (CDCl_3_) δ: 2.37 (3H, s, CH_3_-), 6.82 (1H, s, H-3), 7.17 (1H, dd, *J*_6,5_ = 8.7 Hz, *J*_6,8_ = 2.1 Hz, H-6), 7.42 (1H, d, *J*_8,6_ = 2.1 Hz, H-8), 7.53 (3H, m, H-3′, H-4′, H-5′), 7.91 (2H, dd, *J*_2′,3′(6′,5′)_ = 7.4 Hz, *J*_2′,6′_ = 2.3 Hz, H-2′, H-6′), 8.25 (1H, d, *J*_5,6_ = 8.7 Hz, H-5); ^13^C NMR (CDCl_3_) δ: 21.3 (CH_3_-), 107.8 (C-3), 111.2 (C-8), 119.5 (C-6), 121.9 (C-10), 126.4 (C-2′, C-6′), 127.2 (C-5), 129.2 (C-3′, C-5′), 131.6 (C-1′), 131.8 (C-4′), 154.7 (C-9), 156.8 (C-7), 163.8 (C-2 ), 168.7 (>C = 0) and 177.8 (C-4).

7-Butyryloxyflavone (7-BF, C_19_H_16_O_4_): R_t_ 21.96 min (HPLC); purity 98% (HPLC). ^1^H NMR (CDCl_3_) δ: 1.07 (3H, t, *J* = 7.4 Hz, CH_3_CH_2_CH_2_-), 1.82 (2H, m, CH_3_CH_2_CH_2_-), 2.61 (2H, t, *J* = 7.4 Hz, CH_3_CH_2_CH_2_-), 6.81 (1H, s, H-3), 7.16 (1H, dd, *J*_6,5_ = 8.7 Hz, *J*_6,8_ = 2.1 Hz, H-6), 7.41 (1H, d, *J*_8,6_ = 2.1 Hz, H-8), 7.53 (3H, m, H-3′, H-4′, H-5′), 7.90 (2H, m, H-2′, H-6′), 8.24 (1H, d, *J*_5,6_ = 8.7 Hz, H-5); ^13^C NMR (CDCl_3_) δ: 13.8 (CH_3_CH_2_CH_2_-), 18.5 (CH_3_CH_2_CH_2_-), 36.3 (CH_3_CH_2_CH_2_-), 107.8 (C-3), 111.2 (C-8), 119.6 (C-6), 121.8 (C-10), 126.4 (C-2′, C-6′), 127.2 (C-5), 129.2 (C-3′, C-5′), 131.7 (C-1′), 131.8 (C-4′), 154.8 (C-9), 156.8 (C-7), 163.8 (C-2 ), 171.5 (>C = 0), and 177.8 (C-4).

### 4.3. Biological Methods

#### 4.3.1. Reagents

Soluble recombinant human TRAIL (rhsTRAIL) was purchased from PeproTech Inc. (Rocky Hill, NJ, USA). DMSO (dimethylsulfoxide), DMF (dimethylformamide) and Triton X-100 were obtained from Sigma Chemical Company (St. Louis, MO, USA). 5-Hydroxyflavone, 6-methoxyflavone, 6-Hydroxyflavone, 7-methoxyflavone and 7-Hydroxyflavone were purchased from Sigma Chemical Company. 6-Hydroxyflavone and 7-Hydroxyflavone were obtained by biotransformation of 6-methoxyflavone, 7-methoxyflavone, 6-Hydroxyflavone and 7-Hydroxyflavone. The flavones were dissolved in DMSO (≤50 mM) or in DMF (50 mM) to a final concentration of 0.1% (*v/v*) in culture media.

#### 4.3.2. Cell Culture

The experiments were performed on SW480 and SW620 human colon cancer cells obtained from ATCC (American Type Culture Collection, Manassas, VA, USA). The SW480 and SW620 cells were grown in Leibovitz’s L-15 medium supplemented with 10% heat-inactivated fetal bovine serum, 100 U/mL penicillin and 100 μg/mL streptomycin at 37 °C in a humidified atmosphere of 100% air [45,46]. The reagents for the cell culture were purchased from ATCC or PAA Laboratories (Pasching, Austria).

#### 4.3.3. Detection of Cell Death Using the MTT Colorimetric Assay

Cytotoxicity was determined by the 3-(4,5-dimethylthiazol-2-yl)-2,5-diphenyltetrazolium bromide (MTT) assay as previously described [50,65,66]. SW480 cells (5 × 10^5^/mL) and SW620 cells (2.5 × 10^5^/mL) were seeded 24 h before the experiments in a 96-well plate. Flavones (50–100 μM dissolved in DMF) and/or TRAIL (25–200 ng/mL) were added to the cells for 48 h. After incubation, the medium was removed, and 20 μL MTT solution prepared at 5 mg/mL (Sigma Chemical Company) were added to each well for 4 h. The resulting crystals were dissolved in DMSO. Controls included native cells and medium alone. The spectrophotometric absorbance of each well was measured using a microplate reader (Eon^TM^ Microplate Spectrophotometer, BioTek, Winooski, VT, USA) at 550 nm. The percent cytotoxicity was calculated using the formula: percent cytotoxicity (cell death) = [1 − (absorbance of experimental wells/absorbance of control wells)] × 100%.

#### 4.3.4. Lactate Dehydrogenase Release Assay

Lactate dehydrogenase (LDH) is a stable cytosolic enzyme released from necrotic cells upon membrane damage. The measurement of LDH activity was performed using a commercial cytotoxicity assay kit (Roche Diagnostics GmbH, Mannheim, Germany). LDH is detected in culture supernatants using a coupled enzymatic assay, resulting in the conversion of a tetrazolium salt into a red formazan product. SW480 and SW620 cells were treated with various concentrations of flavones (50–100 μM dissolved in DMF) and/or TRAIL (25–200 ng/mL) for the indicated period of time. The sample solution (supernatant) was removed, and the LDH released from the cells was measured in the culture medium. The maximal release of LDH (positive control) was obtained after treating cells with 1% Triton X-100 for 10 min at room temperature. The spectrophotometric absorbance was measured at 490 nm using a microplate reader (EL x800, Bio-Tek Instruments Inc., Winooski, VT, USA) [52,66]. The necrotic percentage was expressed using the formula: (sample value/maximal release) × 100%.

#### 4.3.5. Determination of Apoptosis by Flow Cytometry with Annexin V-FITC Staining

Apoptosis was determined by flow cytometry using the Apoptest-FITC Kit with annexin V (Dako, Glostrup, Denmark). SW480 cells (5 × 10^5^/mL) and SW620 cells (2.5 × 10^5^/mL) were seeded in 24-well plates for 24 h prior to experimentation and then exposed to flavones (50–100 μM dissolved in DMF) and/or TRAIL (25–200 ng/mL) for 48 h. After incubation, the cells were washed twice with phosphate-buffered saline solution (PBS) and resuspended in 500 μL of binding buffer. The cell suspension (290 μL) was then incubated with 5 μL of annexin V-FITC and 5 μL of propidium iodide for 10 min at room temperature in the dark. The population of annexin V-positive cells was evaluated by flow cytometry (BD LSR II Analyzer, Becton Dickinson Immunocytometry Systems, San Jose, CA, USA) [49,66].

### 4.4. Statistical Analysis

The results are expressed as the means ± SD obtained from three or four separate experiments performed in duplicate (*n* = 6 or *n* = 8) or quadruplicate (*n* = 12). Statistical significance was evaluated using Student’s t test. *p* < 0.05 was considered significant.

## 5. Conclusions

In summary, we synthesized a series of novel flavone derivatives and examined their anticancer effect alone and in combination with TRAIL. The compounds displayed low anticancer activity, but in combination with rhsTRAIL, the effect was enhanced. Our study demonstrates the potential use of tested flavones in TRAIL-based anticancer therapy and prevention. However, further in vitro and in vivo investigations are required to explain the mechanism of action of the flavone analogs.

## Supplementary Materials

Supplementary materials can be found at www.mdpi.com/1422-0067/18/6/1211/s1.

## Abbreviations

TRAIL	tumor necrosis factor-related apoptosis-inducing
V-FITC	annexin fluorescence staining ligand
TNF	tumor necrosis factor
LDH	lactate dehydrogenase assay
MTT	3-(4,5-dimethylthiazol-2-yl)-2,5-diphenyltetrazolium bromide
DMSO	dimethylsulfoxide
DMF	dimethylformamide
PBS	phosphate-buffered saline
