# Supplementary material for: Novel Structurally Related Flavones Augment Cell Death Induced by rhsTRAIL
Source: Int J Mol Sci. 2017 Jun 6;18(6):1211. doi: 10.3390/ijms18061211 (PMC5486034; doi:10.3390/ijms18061211)
Supplement: Supplementary file 1 [file ijms-18-01211-s001.pdf]

Supplementary figures:

Figure 1. Cytotoxic effect of flavones on SW480 and SW620 colon cancer cells. Cells were incubated with compounds at the concentrations of 50 μM and 100 μM for 48 h. The values represent the mean±SD of three independent experiments (n = 3). The percentage of cell death was measured using the MTT cytotoxicity assay (*** = $P < 0.001$ compared to control without flavone, $^{\#} = P < 0.05$, $^{\#\#} = P < 0.01$ and $^{\#\#\#} = P < 0.001$ compared to substrate – 5-HF or 6-HF or 7-HF). Cytotoxic activity of (**A**) 5-HF, 5-AF and 5-BF, (**B**) 6-HF, 6-AF and 6-BF, (**C**) 7-HF, 7-AF and 7-BF against colon cancer cells.

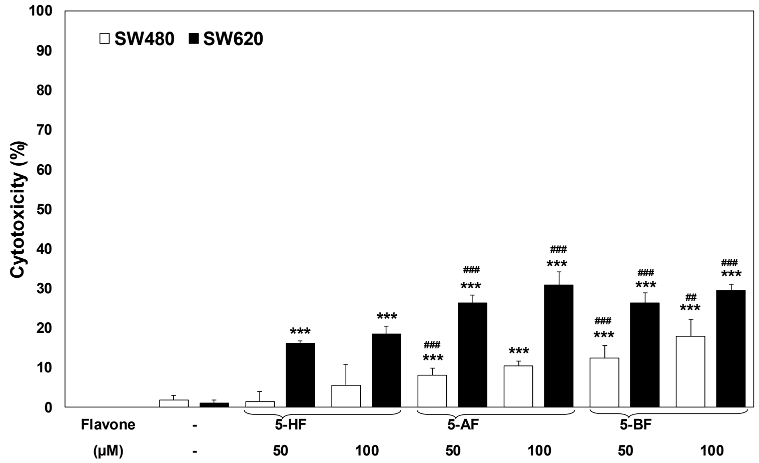

(**A**)

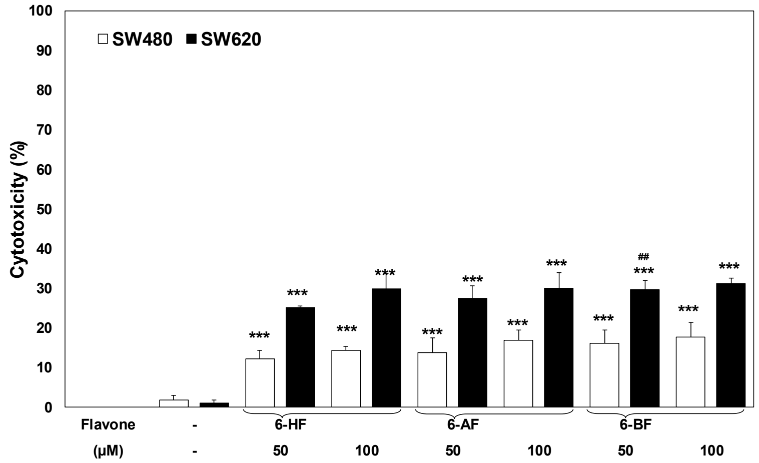

(**B**)

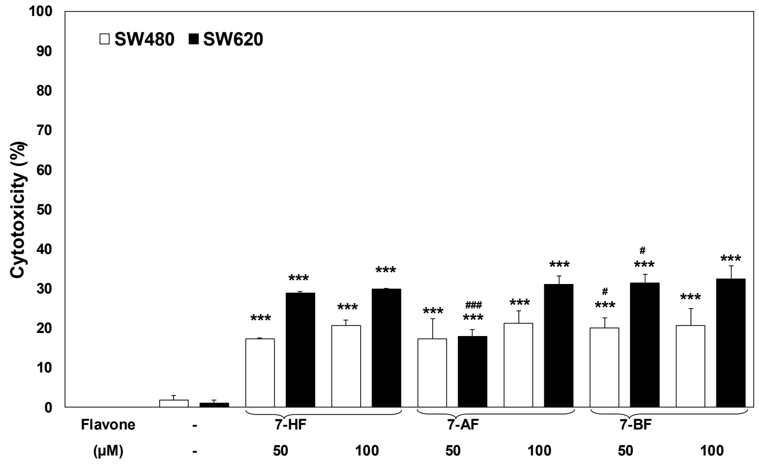

(C)

Figure 2. Apoptotic effect of flavones on SW480 and SW620 colon cancer cells. Cells were incubated with compounds at the concentrations of 50 μM and 100 μM for 48 h. The values represent the mean±SD of three independent experiments (n = 3). Apoptotic cell death was detected by flow cytometry using annexin V-FITC staining (* = $P < 0.05$ and *** = $P < 0.001$ compared to control without flavone, $^{\#} = P < 0.05$, $^{\#\#} = P < 0.01$ and $^{\#\#\#} = P < 0.001$ compared to substrate – 5-HF or 6-HF or 7-HF). Apoptotic activity of (**A**) 5-HF, 5-AF and 5-BF, (**B**) 6-HF, 6-AF and 6-BF, (**C**) 7-HF, 7-AF and 7-BF against colon cancer cells.

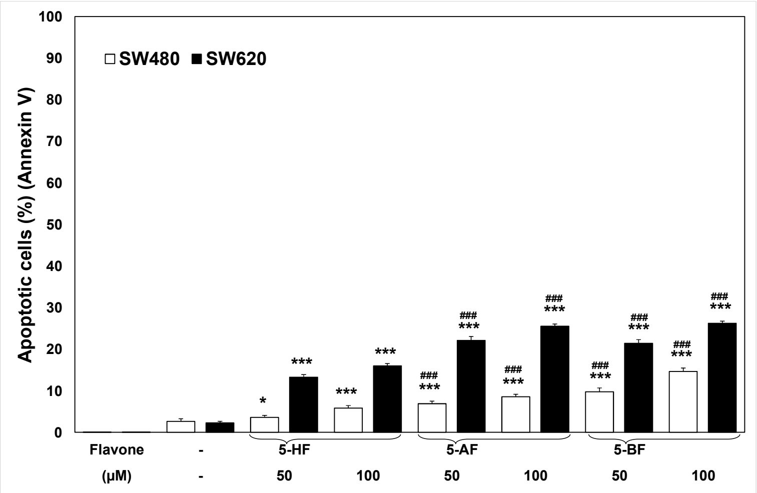

(**A**)

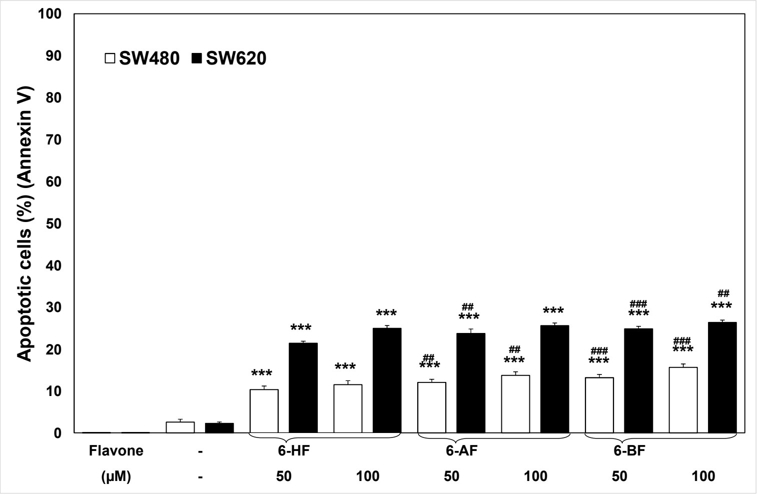
SW480
SW620
Apoptotic cells (%) (Annexin V)
Flavone
(μM)
6-HF
6-AF
6-BF
50
100

(**B**)

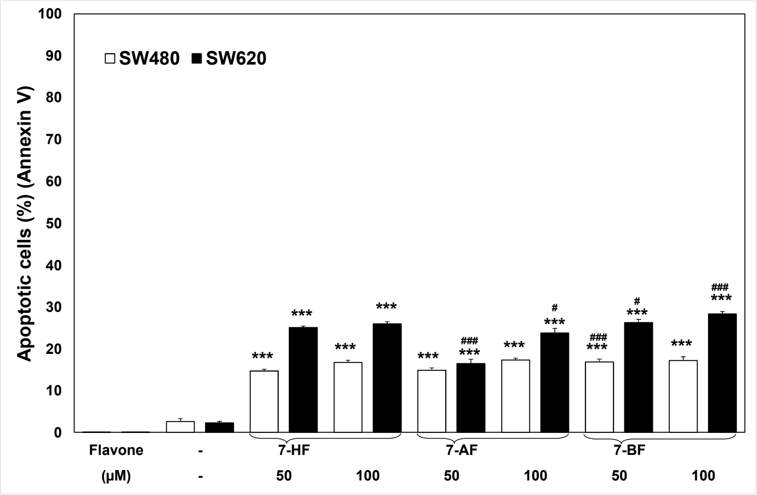
SW480
SW620
Apoptotic cells (%) (Annexin V)
Flavone
(μM)
7-HF
7-AF
7-BF
50
100

(**C**)
